# Antimicrobial Activity of Zinc Oxide Nanoparticles and *Allium Sativum* Extract Against Pathogenic *Escherichia Coli* and *Staphylococcus Aureus* Isolates

**DOI:** 10.24248/eahrj.v9i2.862

**Published:** 2025-12-24

**Authors:** Fromence Nyambu Mwachofi, Anthony Mwangi, Catherine Macharia, Anthony Kebira Nyamache

**Affiliations:** a Department of Biochemistry Microbiology & Biotechnology, Kenyatta University, Kenya; b National Phytotherapeutics Research Centre, Kenyatta University

## Abstract

**Background:**

The increasing trends in antimicrobial resistance (AMR) have continued to pose a global public health concern. This rapid emergence and spread of antimicrobial resistance have necessitated the exploration of innovative approaches to combat microbial infections. This study investigated the potential of enhancing antimicrobial properties through the synergistic effect of zinc oxide nanoparticles (ZnO NPs) and *Allium Sativum* (garlic) extracts against *Escherichia Coli* and *Staphylococcus aureus*.

**Methods:**

Zinc oxide nanoparticles (ZnO NPs), were synthesised using the sol-gel method and later synergised with extracts of Allium sativum. Formation of ZnO NPs was confirmed using X-ray diffraction (XRD), Fourier Transform Infrared Spectroscopy (FTIR) and UV-visible spectrophotometry methods. Similarly, these methods were also used in formed *Allium Sativum* extracts as well as in ZnO NPs and garlic acid combined product. The antimicrobial activity of the nanoparticles against *Staphylococcus aureus* and *Escherichia coli* isolates were determined using Kirby Bauer disc and well diffusion methods.

**Results:**

The average size of ZnO NPs in the present study is 40.96nm with a percentage crystallinity of 58.30%. The minimum inhibitory concentrations (MIC) of ZnO NPs ranged from 5 to 0.312 mg/ml. The antimicrobial assay of both ZnO NPs and crude *Allium Sativum* extracts showed a concentration-dependent effect. The zones of inhibition for ZnO NPs ranged from 18mm to 20mm±2.5, crude *Allium Sativum* extracts 8mm to 14.2mm±2, while the synergistic effect of ZnO NPs and crude *Allium Sativum* extracts was more effective with zones of inhibition ranging from 40mm±2 and 42mm±3.5 against Enteropathogenic *Escherichia coli* and *Staphylococcus aureus*, respectively. Antimicrobial assays revealed that the drug combination exhibited a significantly enhanced inhibitory effect against tested isolates by 35% with reduced minimum inhibitory concentrations (MICs) by 50% compared with individual treatments.

**Conclusion:**

Our findings confirm the promising use of zinc oxide nanoparticles (ZnO NPs) in treating pathogenic *Staphylococcus aureus* and Enteropathogenic *Escherichia coli* (EPEC) bacteria that zinc oxide nanoparticles (ZnO NPs) could be a possible alternative antibiotic. These results highlight the powerful potential of combining nanotechnology with natural products as alternative antimicrobial agents.

## BACKGROUND

Antimicrobial resistance (AMR) continues to be a significant global health threat, affecting healthcare systems, agriculture, and environmental sustainability.^1^ The misuse and overuse of antimicrobials have contributed to the rapid emergence of resistant strains in bacteria, fungi, and viruses, thereby reducing the effectiveness of available treatments.^2^ The World Health Organization (WHO) has raised alarms that AMR could undo decades of medical progress, potentially ushering in a “post-antibiotic era” where even minor infections could become life-threatening.^3^ Specifically, antimicrobial resistance in Enteropathogenic *Escherichia coli* (EPEC) is becoming increasingly problematic, especially in developing regions where EPEC is a leading cause of diarrhoea among infants.^4^ Similarly, *Staphylococcus aureus* represents a major global health threat, particularly due to its widespread resistance to methicillin. The rise of multidrug-resistant (MDR) EPEC and *Staphylococcus aureus* further complicate treatment strategies in these countries, creating a substantial public health risk. Addressing this urgent public health issue requires innovative approaches to combat microbial resistance. One promising alternative involves the use of nanotechnology, particularly metal oxide nanoparticles.^5^

Zinc oxide nanoparticles (ZnO NPs) have gained attention for their exceptional physicochemical properties, including high stability, biocompatibility, and the ability to generate reactive oxygen species (ROS), making them potential solutions.^6–7^ These ROS can disrupt bacterial cell membranes and interfere with cellular functions, rendering ZnO NPs effective against a variety of pathogens, including multidrug-resistant strains.^8^ Unlike conventional antimicrobials, ZnO NPs can target multiple bacterial mechanisms simultaneously, thereby decreasing the likelihood of resistance development.^8–9^ Various medicinal plants have also demonstrated antimicrobial properties. Garlic (*Allium sativa*), used for centuries for its therapeutic benefits, is known for its antimicrobial, antioxidant, and anti-inflammatory properties.^10^

Bioactive compounds in Allium sativum, such as allicin and various organosulfur compounds, exhibit significant antibacterial activity by inhibiting vital bacterial enzymes and disrupting cell wall synthesis.^11^ Importantly, *Allium sativum* preferentially targets pathogenic or harmful microbes while sparing beneficial ones, enhancing its therapeutic potential.^11^ Nanoparticles are increasingly considered alternatives to traditional antibiotics, yet the synergistic effects of combining plant extracts with nanoparticles to enhance their antimicrobial efficacy remain underexplored. This study hypothesises that combining ZnO NPs with *Allium sativum* extract could amplify their individual antimicrobial activities. We investigated the combined effects of zinc oxide nanoparticles and *Allium sativum* extract to determine their capacity to enhance antibacterial properties. The study assessed their antimicrobial activities against *Escherichia coli* and *Staphylococcus aureus* clinical isolates utilising the Kirby Bauer disc diffusion method.

## MATERIALS AND METHODS

This study employed a laboratory-based in vitro experimental study design. It included the synthesis and characterisation of zinc oxide nanoparticles (ZnO NPs), the extraction of phytochemicals from *Allium sativum*, the formulation of a synergistic ZnO–garlic composite, and the assessment of their antibacterial activities against *Escherichia coli* ATCC 25922 and *Staphylococcus aureus* ATCC 25923 through standardised microbiological assays.

### Synthesis of Zinc Oxide Nanoparticles

The nanoparticles were synthesised via the sol-gel method utilising Zinc acetate dihydrate (Zn (CH3COO)2, 2H_2_O, as the precursor and citric acid as the stabiliser agent. Sodium hydroxide served both as a capping agent and to regulate the reaction pH within the range of 8 to 12. Initially, 2g of zinc acetate was dissolved in 15ml distilled water, followed by the addition of 100 mL of ethanol. This solution was heated to 70 degrees Celsius while 0.5M Sodium hydroxide was added slowly to manage nanoparticle aggregation and size dispersion. After gelation, 20ml of 0.5M citric acid was added to aid in nanoparticle chelation and stabilisation. The mixture was kept at constant stirring using a magnetic stirrer for 6 hours. The gel was then allowed to age for 24 hours before being centrifuged at 10,000 rpm for 20 minutes, resulting in a homogeneous and transparent sol. The gel was dried at 270ºC, producing white powdered nanoparticles. These nanoparticles were then calcined at 550 ºC using a muffle furnace and stored in a cool, dry place for further analysis.^12^

### Plant Collection and Preparation of Plant Extracts

Fresh, healthy and mature *Allium sativum* plant; *Allium sativum* var. sativum (soft neck garlic; Artichoke garlic) were obtained from Ruiru sub-county, in Kiambu, Kenya. The plants that were collected were treated as previously described by washing them twice with distilled water and then allowing them to air dry at room temperature.^13^

The cloves were crushed using a mortar and pestle and then immediately subjected to methanolic extraction. Methanol was added in a 2:1 ratio (two parts solvent to one part of *Allium sativum*), and the mixture was allowed to settle while being stirred periodically over a five-day period in a dark environment to enhance extraction. Following this extraction phase, the mixture was filtered through filter paper with the assistance of a vacuum pump to separate the garlic extract from any solid residues. The resulting garlic extract, which exhibited a pale-yellow colour, was concentrated using a rotary evaporator. Finally, the crude extract was stored in a refrigerator at 9ºC for future analysis.^13^

### Synergy of ZnO NPs with Allium Sativum Extract

The synergy between zinc oxide nanoparticles and *Allium sativum* extract was achieved through post-synthesis modification of the nanoparticles. The synthesis of the nanoparticles was done using the sol-gel method with *Allium sativum* extract added to the gel to functionalise them. The mixture was vortexed for 20 minutes and then subjected to ultrasonication at 25 °C for an additional 20 minutes. This process was repeated six times to guarantee proper aggregation and dispersion of the compounds. The final product was subsequently refrigerated at 9 °C for further analysis.^14^ The use of *Allium sativum* var. sativum was based on its high abundance and multiple bioactive compounds, while ZnO NP was due to its stability, surface functionalisation and antimicrobial synergy.

### Liquid Chromatography - Mass Spectrometry

An LCMS scan was performed using (Shimadzu 8040 LCMS from Japan) to analyse the crude *Allium sativum* extract for the presence of Allicin as previously described.^20^ The analysis utilised two scan modes; a full scan and a targeted Multiple Reaction Monitoring (MRM), with Allicin as the specific target molecule. The MRM parameters used comprised a precursor ion of 163.0 and a daughter ion of 73.0, with a collision energy (CE) of −35 V^19^. For the LCMS analysis, a Kinetex 2.6 μm XB-C 18 column (150mm x 3mm) was used, along with a Phenomenex Security Guard Ultra cartridge as the guard column. The mobile phase comprised 0.1% formic acid in water (A) and acetonitrile (B), following a gradient program: from 0 to 0.5 min, 10% B; from 0.5 to 5 min, 10% to 50% B; from 5 to 8 min, 50% to 90% B; from 8 to 11 min, 90% B; from 11.0 to 11.01 min, 90% to 10% B; from 11.01 to 20.0 min, 10% B. The flow rate was maintained at 0.25 mL/min, and the oven temperature was kept at 40 °C. A 5 μl injection volume was utilised, and the mass spectrometry interface operated in Electro-Spray Ionisation (ESI) mode under MRM conditions. Nitrogen gas was employed both as a nebulising gas at a flow rate of 3 L/min and as a drying gas at a flow rate of 15 L/min. The MS temperature settings included a desolvation line temperature of 250 °C and a heating block temperature of 400 °C.^20^

### Antimicrobial Activity

The antimicrobial activity of ZnO NPs, *Allium sativum* explants and combined synthesised ZnO NPS and *Allium sativum* extracts were evaluated against standard strains (*Escherichia coli* ATCC 25922 and *Staphylococcus aureus* ATCC 25923 strains as previously described in agar well diffusion methods.^15–16^ The wells of 6mm diameter were punched into Muller Hinton agar having the test organisms (5 × 10^5^ CFU/ml). The wells were filled with 100 μl of ZnO NPS (5, 2.5, 1.25, 0.625, 0.312 and 0.156 mg/ml). Similarly, to *Allium sativum* plant extracts and in combinations (synergistic). Gentamycin (10 μg), (30 μg), co-trimoxazole (25 μg), tetracycline (30 μg), chloramphenicol (50 μg), kanamycin (30 μg), streptomycin (10 μg), Ampicillin (25 μg) and sulfamethoxazole (20μg) (Oxoid) were used as controls for both Gram-negative and Gram-positive. The inoculated plates were then incubated at 37°C for 18 to 24 hours. The antimicrobial activity was then determined by measuring the zones of inhibition against the test organisms in millimetres.^15–18^

### The X-ray Diffraction (XRD)

The X-ray Diffraction (XRD) analysis of ZnO Nanoparticles was conducted as previously described using an X-ray Diffractometer (Equinox 100, from Thermofischer). 21 This XRD analysis was performed to confirm the crystal structure, particle size, purity, and crystallinity of the ZnO nanoparticles known have impact on their antimicrobial activity.^22^ The observations were done and recorded over an energy range of 0 to 5000 at a scan rate of 20/min. Cu Alpha radiation (λ=1.5406 Å) was used with an accelerating voltage of 40 kV.^21^ The diffraction patterns were measured at 2θ (diffraction angle) for structural properties characterisation of the nanoparticles. The Scherrer's Equation was used to calculate the size of nanoparticle. An online software was used {XRD Crystallite (grain) Size Calculator (Scherrer Equation) - InstaNANO. https://instanano.com/all/characterisation/xrd/crystallite-size/ (accessed April 16th, 2024).

The Scherrer Equation used:


$$\text{D}=\frac{\text{Kλ}}{\text{βcosθ}}$$

D=The nanoparticle size (Crystallite size)

K=0.9 (Scherrer constant)

λ=0.15406 nm (wavelength of the x-ray sources)

β=FWHM (in radians) (calculated to 0.20928)

θ=Maximum intensity peak position. (at 28.88°)

### UV-Vis Spectrophotometry

The absorbance of the samples (synergised zinc oxide nanoparticles or *Allium sativum* plant extracts) was measured for its maximum absorbance using UV-Vis spectrophotometry (Shimadzu 1900i UV-Vis spectrophotometer, Japan). The optical property of ZnO nanoparticles was analysed via an ultraviolet and visible absorption spectrophotometer at the wavelength range of 300 to 600 nm. This analysis was done with the aim of discerning any changes in absorbance compared to the individual compounds.^23^

### Fourier Transform Infrared Refractometer (FTIR)

The binding properties of ZnO nanoparticles using *Allium sativum* (garlic) extracts were determined using Fourier Transform Infrared Refractometer (FTIR) Spectroscopy analysis (Shimadzu QATR-S from Japan). To analyse the functional groups and chemical composition of the nanoparticles, dried powder of the synthesised ZnO nanoparticles as well as the *Allium sativum* was measured at percentage transmittance (%T) over a wavelength range of 400 to 4000 cm-1 with the resolution of 4cm-1.^24, 25, 26^

### Data Analysis

The inhibition zone diameter data were analysed using one-way analysis of variance (ANOVA). The differences were considered significant at *P* value <.05.

## RESULTS

### Liquid Chromatography-Mass Spectrophotometry Analysis

**FIGURE 1:**
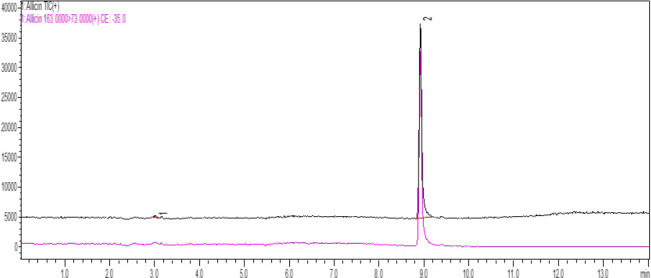
Chromatogram Showing Allicin Eluting at 8.932 Minutes

Characterisation of active target compounds in the plant extracts was done using Liquid Chromatography-Mass Spectrometry (LC-MS). The analysis of the presence of the antimicrobial compounds yielded a pale-yellow extract, confirming the presence of allicin, the target compound. When the extracts were subjected to liquid chromatography analysis, it confirms the specific transitions of precursor and daughter ions. The precursor ion at 163.0 m/z and the daughter ion at 73.0 m/z monitors detections, confirms the presence of allicin compound. The elution of allicin occurred at 8.932 minutes, further confirms allicin in presence in the crude *Allium sativum* extract (Figure 1).

### Antibacterial Activity of ZnO NPs

The biosynthesised ZnO nanoparticles using methanolic *Allium sativum* extracts demonstrated significant antibacterial activity against the bacterial strains tested: enteropathogenic *Escherichia coli* ATCC 25922 and *Staphylococcus aureus* ATCC 25923. The results indicated that the inhibition zone diameter of ZnO nanoparticles were varied from 10 to 18 mm for enteropathogenic *Escherichia coli* and from 12 to 20 mm for *Staphylococcus aureus.*

The low concentration of *Allium sativum* extracts or ZnO NPS that had the most effective (large zone of inhibition) were 5 mg/ml for ZnO NPs and 0.33 mg/ml for *Allium sativum* extracts. The synergistic activity was also performed with higher zones of inhibition being shown. From the analysis, the minimum concentration of 5 mg/ml for ZnO NPs while *Allium sativum* extracts was 0.33 mg/ml. These were lowest concentrations that were able to inhibit bacterial growth. According to the one-way ANOVA analysis, there was a significant difference between ZnO nanoparticles and *Allium sativum* extracts against tested isolates (*p*>.011). The Zinc Oxide nanoparticles demonstrated significant higher antimicrobial activity than - *Allium sativum* extracts. When the ZnO NPs and *Allium sativum* were used in combination much higher significant antimicrobial activity was observed (*p*>.001) confirming the synergistic activity (Table 1).

**TABLE 1:** The average minimum inhibitory concentration of Allium sativum extracts, Zinc oxide nanoparticles and combined (synergistic) against Escherichia coli and Staphylococcus aureus

Bacterial Strain	Crude Garlic Extract	ZnO NPs	(ZnO NPs + Garlic Extract)	Control Antibiotics
*E. coli*	8	10	30	30
*E. coli*	10	18	26	26
*E. coli* ATCC 25922	12	17	28	28
*S. aureus*	12	18	32	32
*S. aureus*	10	16	40	40
*S. aureus* ATCC 25923	14	15	24	24
*P* value		*p*>.0011	*p*>.0001	

### Biosynthesis and Characterisation of ZnO NPs

**FIGURE 2:**
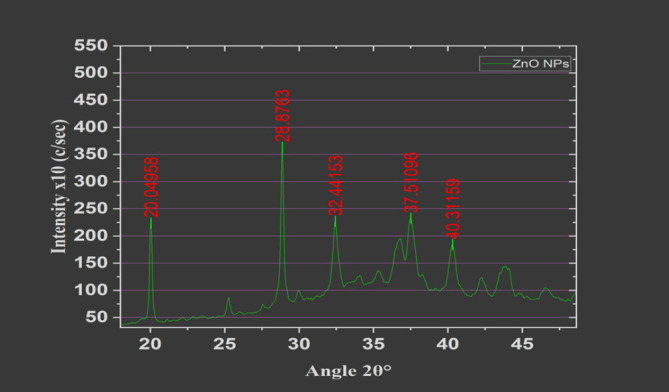
XRD patters of synthesized ZnO NPS

The X-ray diffraction (XRD) analysis revealed distinct peaks corresponding to the crystallographic planes of zinc oxide (ZnO) with a wurtzite crystal structure. The synthesised ZnO nanoparticles from the Scherrer equation analysis, confirmed an estimated average crystallite size, with size of 40.96 nm. The percentage crystallinity was calculated at 58.34%, which was derived from the ratio of the area of crystalline peaks to total peak area.^28^ The maximum intensity peak was observed at 28.88°, with other notable peaks aligning well with expected positions for various planes of ZnO (JCPDS #03-065-3411). The absence of unidentified peaks indicated the high sample purity.^27^ The sharpness of the peaks confirmed the crystallinity of the synthesised ZnO nanoparticles (NPs). However, in this analysis, a slight shifting and broadening of some peaks were observed which could indicate a the effect of potential strain or doping effects observed within the crystal lattice, which could warranting further investigation (Figure 2).

Nanoparticles using methanolic Allium sativum extracts at the range of 20 from 10 to 80 with a scan rate of 20/min utilizing Cu Alpha (lambda = 1.5406A) radiation with an accelerating voltage of 40 kV. Different stages present in the samples were assessed by means of X-pert high score programming having a search match office. The maximum intensity peak occurred at 28.88°.

### UV-Visible Spectrometry

**FIGURE 3:**
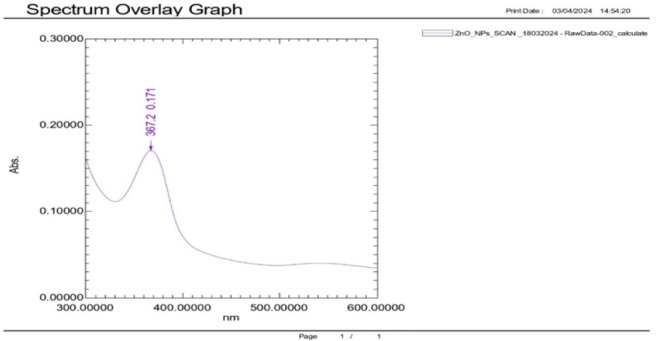
UV-Visible spectrometry for ZnO NPS

The UV-Vis spectroscopy analysis of the biosynthesised Zinc Oxide (ZnO) nanoparticles, methanolic garlic extracts, and their combination showed a unique absorption spectrum. For instance, the absorption spectra of antimicrobial ZnO nanoparticles was within the 300 to 400 nm range, with distinct absorption peak at 367.2nm with absorbance of 0.171. The peak at 367.2nm is typical peak for ZnO nanoparticles hence confirming the successful synthesis of ZnO nanoparticles at 5 mg/m based on its direct band gap (∼3.3 eV), which is within the UV absorption range of ∼320 to 380 nm. This absorption peak confirms the ZnO good crystalline quality and quantum size effects. The obtained moderate absorbance of 0.171 suggest the active particle activity at lowest concentration or minimum agglomeration based on the methods used. An activity at lower concentration which could be used. The absorption peak at 367.2nm However, when compared to the ZnO + *Allium sativum* formulation, the shift in peak position and increased absorbance indicates significant surface interaction and enhancement due to the garlic extract (Figure 3).

Concentration of 5mg/ml revealing a peak at 367.2nm

**FIGURE 4:**
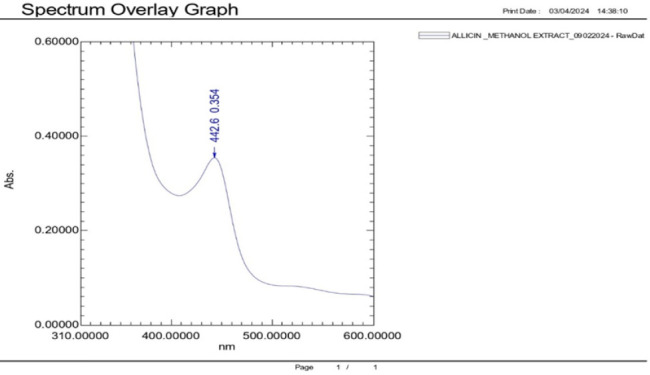
Visible spectrometry of *Allium sativum*

Similarly for *Allium sativum* extracts at the concentration of 0.33 mg/mL, the UV-Visible Spectroscopy profile analysis showed an absorption peak at 442.6 nm with an absorbance of approximately 0.354. This absorption falls within chromophores absorption range 400 to 450nm, potentially linked to compounds found in *Allium sativum* extracts. The peak at 442.6 nm indicates the presence of UV-active phytochemicals, mainly sulfur-containing compounds and potentially phenolics. These may include organosulfur compounds like allicin, ajoene, or diallyl sulfides. The peaks confirm the existence of active phytochemical constituents (Figure 4).

**FIGURE 5:**
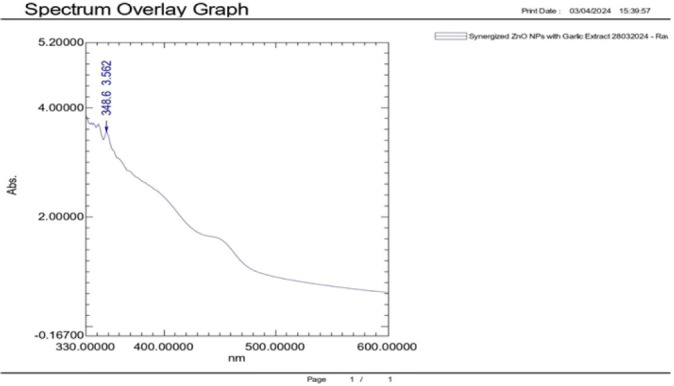
UV-Visible Spectroscopy Analysis of Combined Synergized ZnO Nanoparticles (NPs) and *Allium Sativum* Extracts Lowest Concentration of 5mg/ml Reveal a Prominent Peak at 348.6 nm

In the formulation of ZnO nanoparticles (NPs) combined with *Allium sativum* extract at a concentration of 5 mg/mL, UV-Visible Spectroscopy analysis indicated a significant absorption peak at 348.6 nm, with an absorbance of 3.562. This peak at 348.6 nm suggests the presence of ZnO nanoparticles, which generally absorb in the UV range of approximately 320 to 380 nm due to an intrinsic band gap of around 3.3 eV. This observation supports the successful synthesis of ZnO nanoparticles and their optical stability within the garlic extract matrix. Additionally, the absorption shift from 367.2 nm in ZnO alone to 348.6 nm in the ZnO-garlic nanocomposite indicates a blue shift. This shift may be associated with the surface modification of ZnO by garlic phytochemicals, potentially affecting particle size, surface energy, or resulting from quantum confinement effects, leading to the formation of more stabilised nanoparticles (Figure 5).

### Fourier Transform Infrared Spectroscopy

**FIGURE 6:**
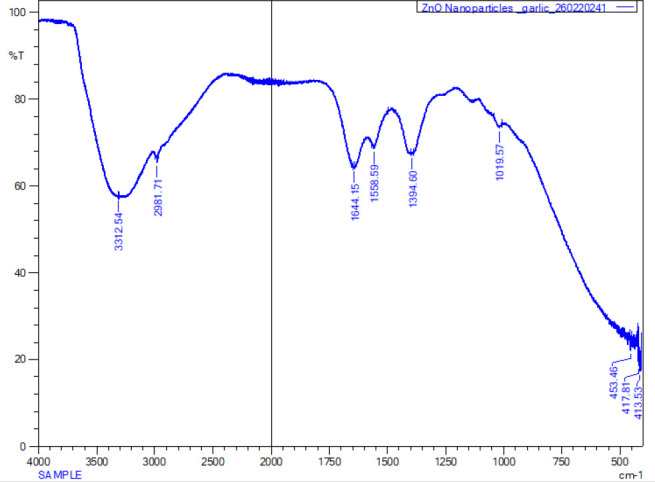
FTIR Spectroscopy of synergized ZnO NPs with a Crude *Allium Sativum* Extract

The FTIR spectrum reveals clear absorption peaks that are associated with different functional groups derived from both the phytochemicals present in the *Allium sativum* extract and the ZnO nanoparticles. These FTIR absorption peaks that occurred at 453.46, 1019.57, 1394.60, 1558.59, 1644.15, 2981.71, and 3312.54 cm-^1^ corresponded with different functional groups. The FTIR absorption spectrum revealed several broad band peaks with a prominent band observed at 3312.54 cm^−1^ that is around 3200–3400 cm^−1^ which corresponds to O–H stretching vibrations of hydroxyl groups possibly from alcohols and phenols. A peak at 2981.71 occurred in the region of 2900 cm^−1^ representing C–H stretching vibrations from alkanes indicative of the presence organic compounds from plant extracts. Additionally, a strong peak occurring at 1644.15, which is near 1650-1600 cm^−1^ corresponds to C–O stretching (carbonyl groups) or C=C stretching from aromatic rings. The compound could be originating from flavonoids or sulfur-containing compounds in garlic, suggesting their involvement in nanoparticle stabilisation. Furthermore, another peak was detected at 1394.60 cm^1^ occurring in the range of 1040 cm^−1^ which could be due to C–N stretching or CH bending vibrations, which could be linked to proteins or other indigenous compounds in the plant extracts. However, there was also an occurrence of a strong absorption that occurred at 453.46 which is below 600 cm^−1^ (typically 450–550 cm^−1^). This peak is a characteristic feature of Zn–O stretching vibrations, which confirmed the formation of ZnO nanoparticles (Figure 6).

The peaks show the preservation of the stretches from the functional groups of the individual materials in the mixture (help maintain the structure and boost antibacterial efficacy).

### Infrared (FTIR) Analysis of Biosynthesised ZnO NPs and Methanolic Allium sativum Plant Extracts.

**FIGURE 7:**
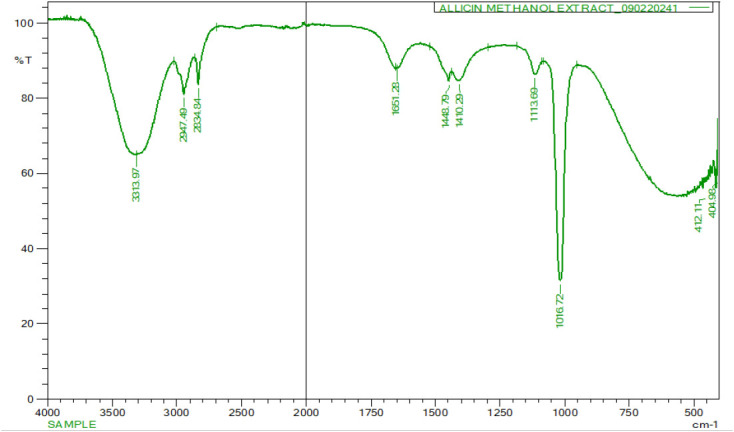
FTIR Spectroscopy of a Crude *Allium Sativum Extract*

The FTIR analysis was performed on the methanolic *Allium sativum* extract to examine the functional groups present on the surface of bio ZnO nanoparticles. The FTIR spectrum of the crude *Allium sativum* typically displayed characteristics absorption bands corresponding to diverse array of functional groups inherent in its phytochemicals. The FTIR spectrum of the crude revealed several broad band peaks with a prominent band observed around 3200–3400 cm^−1^ which is attributed to O–H stretching vibrations associated with alcohols and phenols. A peak in the region of 2920 to 2850 cm^−1^ corresponds to C–H stretching vibrations from aliphatic chains, indicating the presence of various hydrocarbons and organic constituents. Additionally, a strong band near 1650 cm^−1^ was typically associated with C–N stretching or CH bending, suggesting the presence of amines or amino acid derivatives. Furthermore, another peak was detected in the range of 1040 to 1100 cm^−1^ linked to C–O stretching vibrations, suggesting the presence of alcohols, ethers, or esters, indicating the presence of saccharides or glycosidic components. This FTIR profile of crude *Allium sativum* extract, confirms the presence of diverse array of functional groups, that includes; hydroxyls, carbonyls, amines, aliphatic chains and esters at various peaks (Figure 7).

The peaks show the preservation of the stretches from the functional groups of the individual materials in the extracts

## DISCUSSION

This study evaluated the antibacterial efficacy of zinc oxide nanoparticles (ZnO NPs), *Allium sativum* extracts, and their synergistic combination against *Escherichia coli* and *Staphylococcus aureus* isolates. Consistent with previous studies both ZnO NPs and *Allium sativum* extracts displayed significant antibacterial activity when used separately or in combination^29^. The ZnO NPs exhibited broad-spectrum antimicrobial activity against both *E. coli* and *Staphylococcus aureus* isolates. This findings could be attributed to their nanoscale size, high surface area, and ability to generate reactive oxygen species (ROS) and induce oxidative stress which can disrupt bacterial membranes. In addition, the release of Zn^2+^ ions further enhances antimicrobial activity by destabilising bacterial membranes and inhibiting enzyme function.

Conversely, *Allium sativum* extracts, had antimicrobial activities against the tested isolates, confirming their antimicrobial potential. This effect is largely associated with allicin and other sulfur-containing compounds, which modify thiol groups in key bacterial enzymes, leading to metabolic disruption and impaired cellular function.^30^ Interestingly, both *Allium sativum* extracts and ZnO nanoparticles exhibited increased antimicrobial activity at lower concentrations, a phenomenon that may be linked to their antioxidant and antimicrobial properties. Overall, ZnO nanoparticles demonstrated greater antibacterial efficacy than *Allium sativum* extracts alone, likely due to their capacity for sustainable ROS generation and zinc ion release.

In this study, the combination of *Allium sativum* extracts and ZnO nanoparticles produced significantly higher zones of inhibition compared to either agent used independently (Table 1). The finding confirms a synergistic interaction between *Allium sativum* and ZnO NPs emphassing the importance of combined antimicrobial strategies. Similar synergistic enhancements have been reported in studies involving ZnO NPs combined with other medicinal plant extracts.^17^ The observed synergy may be attributed to complementary mechanisms of action, whereby ZnO NPs increase bacterial membrane permeability, facilitating greater uptake of allicin and other bioactive sulfur compounds, while concurrently inducing oxidative damage through ROS production. These combined effect amplify cellular injury and bacterial death.^8, 31^

Therefore, the ZnO NPs and *Allium sativum* agents have demonstrated antibacterial activity efficacy against Gram-negative and Gram-positive bacteria. The green synthesis of ZnO NPs using *Allium sativum* provides a broad mode of action with potential applications beyond antimicrobial therapy, including antifungal, anticancer, and agricultural uses.^35^ However, it is important to note that high concentrations of ZnO NPs or *Allium sativum* extracts may pose cytotoxic risks to mammalian cells. The synergistic formulation allows for reduced nanoparticle dosage while maintaining or enhancing antimicrobial efficacy, thereby minimising potential toxicity.^36–37^ Additionally, *Allium sativum* has been reported to selectively inhibit pathogenic bacteria such as *E. coli* while exerting minimal effects on beneficial microflora, including *Lactobacillus casei*.^38^ This selective activity suggests that *Allium sativum* -based formulations may be microbiota-friendly while effectively targeting pathogenic organisms.^38–40^ The thermal instability of allicin, which limits its antimicrobial efficacy under standard conditions, could also be mitigated through stabilisation via interaction with ZnO NPs. Nanoparticle binding may stabilise allicin, preserve its chemical integrity, and prolong its bioactivity. The antimicrobial properties of *Allium sativum* can further be attributed to its composition, which consists of 33 sulfur compounds, 17 amino acids, enzymes, minerals, vitamins, and valuable essentials^30^.

Additionally, *Allium sativum* has been reported to selectively inhibit pathogenic bacteria such as *E. coli* while exerting minimal effects on beneficial microflora, including *Lactobacillus casei*. This selective activity suggests that *Allium sativum* - based formulations may be microbiota-friendly while effectively targeting pathogenic organisms. The thermal instability of allicin, which limits its antimicrobial effectiveness under standard conditions, may also be mitigated through interaction with ZnO nanoparticles. Nanoparticle binding may stabilise allicin, preserve its chemical integrity, and prolong its bioactivity. The antimicrobial properties of *Allium sativum* can further be attributed to its complex composition, which includes sulfur compounds, amino acids, enzymes, minerals, vitamins, and essential bioactive components.

### Strengths and Limitations of the Study

This study had a number of limitations despite its promising findings. First, the antibacterial activity of ZnO nanoparticles and *Allium sativum* extracts was evaluated in vitro assays, which may not fully reflect the in vivo conditions. Secondly, while synergistic antibacterial effects were clearly demonstrated, the precise molecular mechanisms involving in the interaction between ZnO nanoparticles and garlic-derived bioactive compounds were not fully elucidated.

## CONCLUSION

This study provides compelling evidence for the enhanced antibacterial efficacy of biosynthesised ZnO nanoparticles combined with *Allium sativum* extracts. Compared to individual agents, the synergistic formulation exhibited superior antimicrobial activity, improved stability of bioactive compounds, and reduced potential cytotoxicity. The successful biosynthesis of ZnO NPs using methanolic garlic extracts was confirmed by UV–Vis spectroscopy (λmax at 344 nm), TEM, FTIR, and EDX analyses, with particle sizes ranging from 10 to 80 nm. The strong antibacterial activity observed against clinical isolates of *Staphylococcus aureus* and enteropathogenic *Escherichia coli* highlights the potential of this green nanotechnologybased approach as a promising alternative strategy for combating the escalating challenge of antimicrobial resistance.

## References

[1] Salam, MA, Al-Amin, MY, Salam, MT, et al. Antimicrobial resistance: A growing serious threat for global public health. Healthcare (Basel). 2023;11(13):1946. 10.3390/healthcare11131946 37444780 PMC10340576

[2] Baweja, R, Singh, M, Shukla, S, Ravi, R, Ahmad, R, & Mishra, A. Antimicrobial Resistance: Mechanism, Causes, Prevention and Societal Impact. The Microbe. 2025;9:00617. 10.1016/j.microb.2025.100617

[3] World Health Organization. (2017). Antibacterial agents in clinical development: An analysis of the antibacterial clinical development pipeline. WHO. https://www.who.int/publications/i/item/antibacterial-agents-in-clinical-development Accessed 27 2025

[4] Chellapandi, K, Dutta, TK, Sharma, I, De Mandal, S, Kumar, NS, Ralte, L. Prevalence of multidrug-resistant enteropathogenic and enteroinvasive Escherichia coli isolated from children with and without diarrhea in Northeast Indian population. Annals of Clinical Microbiology and Antimicrobials. 2017;16(1):49. https://doi.org10.1186/s12941-017-0225-x 28693504 10.1186/s12941-017-0225-x PMC5504610

[5] Parvin N, Joo SW, Mandal TK. (2025). Nanomaterial-based strategies to combat antibiotic resistance: Mechanisms and applications. Antibiotics, 14(2): 207. 10.3390/antibiotics14020207. 40001450 PMC11852044

[6] Lebaka VR, Ravi P, Reddy MC, Thummala C, Mandal TK. (2025). Zinc oxide nanoparticles in modern science and technology: multifunctional roles in healthcare, environmental remediation, and industry. Nanomaterials. 2025;15(10):754. 10.3390/nano15100754 40423144 PMC12113910

[7] Mishra PK, Mishra H, Ekielski A, Talegaonkar S, Vaidya B. Zinc oxide nanoparticles: a promising nanomaterial for biomedical applications. Drug discovery today. 2017;22(12):1825–1834. 10.1016/j.drudis.2017.08.006 28847758

[8] Wang, L, Hu, C, Shao, L. (2017). The antimicrobial activity of nanoparticles: Present situation and prospects for the future. International Journal of Nanomedicine, 12, 1227–1249. 10.2147/IJN.S121956 28243086 PMC5317269

[9] Elbehiry, A, Abalkhail, A. Antimicrobial Nanoparticles Against Superbugs: Mechanistic Insights, Biomedical Applications, and Translational Frontiers. Pharmaceuticals. 2025;18(8):1195. 10.3390/ph18081195 40872586 PMC12389178

[10] Verma, T, Aggarwal, A, Dey, P, et al. Medicinal and therapeutic properties of garlic, garlic essential oil, and garlic-based snack food: An updated review. Frontiers in Nutrition. 2023;10:1120377. 10.3389/fnut.2023.1120377 36875845 PMC9978857

[11] Bhatwalkar, SB, Mondal, R, Krishna, SB, Adam, JK, Govender, P, Anupam, R. Antibacterial properties of organosulfur compounds of garlic (Allium sativum). Frontiers in Microbiology. 2021;12:613077. 10.3389/fmicb.2021.613077 34394014 PMC8362743

[12] Mahamuni, PP, Patil, PM, Dhanavade, MJ. Synthesis and characterization of zinc oxide nanoparticles by using polyol chemistry for their antimicrobial and antibiofilm activity. Biochemical and Biophysical Reports. 2018;17:71–80. 10.1016/j.bbrep.2018.11.007 PMC6295600 30582010

[13] Bar, M, Binduga, UE, Szychowski, KA Methods of isolation of active substances from garlic (Allium sativum L.) and its impact on the composition and biological properties of garlic extracts. Antioxidants. 11(7), 2022;1345. https://doi.org10.3390/antiox11071345 35883836 10.3390/antiox11071345 PMC9312217

[14] Khan, SA., Shahid, S, & Lee, CS. Green synthesis of silver nanoparticles using plant extracts and their antimicrobial activities: A review of recent literature. RSC Advances. 2021;11(78):44244–44268. 10.1039/D0RA09941D PMC8694026 35424248

[15] Kebede, A, Kemal, J, Alemayehu, H, Habte MS. Isolation, identification, and antibiotic susceptibility testing of salmonella from slaughtered bovines and ovines in Addis Ababa Abattoir Enterprise, Ethiopia: A cross-sectional study. International Journal of Bacteriology. 2016; 2016:3714785. 10.1155/2016/3714785 27660816 PMC5021890

[16] Hozyen, HF, Ibrahim, ES., Khairy, EA, El-Dek, SI. Enhanced antibacterial activity of capped zinc oxide nanoparticles: A step towards the control of clinical bovine mastitis. Veterinary World. 2019;12(8):1225–1232. 10.14202/vetworld.2019.1225-1232 31641301 PMC6755405

[17] Dobrucka, R, Długaszewska, J. Biosynthesis and antibacterial activity of ZnO nanoparticles using Trifolium pratense flower extract. Saudi Journal of Biological Sciences. 2016;23(4):517–523. 10.1016/j.sjbs.2015.05.016 27298586 PMC4890195

[18] Parvekar, P, Palaskar, J, Metgud, S, Maria, R, Dutta, S. The minimum inhibitory concentration (MIC) and minimum bactericidal concentration (MBC) of silver nanoparticles against Staphylococcus aureus. Biomaterial Investigations in Dentistry. 2020:7(1):105–109. 10.1080/26415275.2020.1796674 32939454 PMC7470068

[19] Ramachandram D, Dinesh, R. LCMS—A review and a recent update. World Journal of Pharmacy and Pharmaceutical Sciences. 2018;7(12):337–362. 10.20959/wjpps20165-6656

[20] Essa, AF., El-Hawary, SS, Kubacy TM, et al. Integration of LC/MS, NMR and molecular docking for profiling of bioactive diterpenes from Euphorbia mauritanica L. with in-vitro anti-SARS-CoV-2 activity. Molecules. 2023;28(1):123. DOI: 10.1002/cbdv.202200918 36602020

[21] Azad MA, Hossain MS, Saikat SP, Hasan MR, Bhowmik S. Structural insights into zinc oxide-silver nanocomposite via different XRD models: rapid synthesis with photocatalytic & antibacterial applications. Nanoscale Adv. 2025;7(24):8138–8153. 10.1039/D5NA00790A 41268045 PMC12628709

[22] Saeed, AA. Structural and Optical Properties for ZnO Nanoparticles for Antibacterial Application. Tikrit Journal of Pure Science. 2025;30(1):62–70. DOI: 10.25130/tjps.v30i1.1779

[23] Ghosh, S, Patil, S, Ahire, M, et al. Gnidia glauca flower extract mediated synthesis of gold nanoparticles and evaluation of its chemocatalytic potential. Journal of Nanobiotechnology. 2012;10(1):17. 10.1186/1477-3155-10-17 22548753 PMC3462129

[24] Ahmed, S, Ahmad, M, Swami, BL., Ikram, S. Green synthesis of silver nanoparticles using Azadirachta indica aqueous leaf extract. Journal of Radiation Research and Applied Sciences. 2016;9(1): 1–7. 10.1016/j.jrras.2015.06.006

[25] Eid, MM. Characterization of nanoparticles by FTIR and FTIR-microscopy. In Handbook of Consumer Nanoproducts. Singapore: Springer; 2022:1–30. DOI: 10.1007/978-981-15-6453-6_89-1

[26] Mohamed, R, & Anuar, MA. Structural and electrochemical behaviors of ZnO structure: Effect of different zinc precursor molarity. Condensed Matter. 2022;7(4):71. 10.3390/condmat7040071

[27] Abdelbaky, AS, Abd El-Mageed, TA, Babalghith, AO, Selim, S, Mohamed, AM. Green synthesis and characterization of ZnO nanoparticles using Pelargonium odoratissimum (L.) aqueous leaf extract and their antioxidant, antibacterial and anti-inflammatory activities. Antioxidants. 2022;11(8):1444. 10.3390/antiox11081444 35892646 PMC9329751

[28] Ju, X, Bowden, M, Brown, EE, Zhang, X. An improved X-ray diffraction method for cellulose crystallinity measurement. Carbohydrate polymers. 2015;123:476–481. 10.1016/j.carbpol.2014.12.071 25843882

[29] Al-Badaii, F, Al-Khalidy, A, Khalid, M, et al. Green synthesis of zinc oxide nanoparticles using Allium Sativum extract: evaluation of antibacterial activity against nosocomial bacteria. Thamar University Journal of Natural & Applied Sciences. 2024;9(1):14–19. 10.59167/tujnas.v9i1.2049

[30] Magryś, A, Olender, A, Tchórzewska, D. Antibacterial properties of Allium sativum L. against the most emerging multidrug-resistant bacteria and its synergy with antibiotics. Archives of microbiology. 2021;203(5):2257–2268. 10.1007/s00203-021-02248-z 33638666 PMC8205873

[31] Abu-Gharbia, MA., El-Sayed, MF, Salem, JM., Abd-Elsamei, WM, Al-Arabi, G. Antibacterial impact of biosynthesized zinc oxide nanoparticles on uropathogenic Escherichia coli and in vivo assessment of physiological and histological alterations. Scientific Reports. 2025;15(1); 5721. 10.1038/s41598-025-98060-6 40325065 PMC12053763

[32] Droepenu, EK., Amenyogbe, E, Boatemaa, MA, Opoku, E. Study of the antimicrobial activity of zinc oxide nanostructures mediated by two morphological structures of leaf extracts of Eucalyptus robusta Sm. Heliyon. 2024;10(4):e25590. 10.1016/j.heliyon.2024.e25590 38370246 PMC10869787

[33] Salem, MS, Mahfouz, AY, Fathy RM. The antibacterial and antihemolytic activities assessment of zinc oxide nanoparticles synthesized using plant extracts and gamma irradiation against the uro-pathogenic multidrug resistant Proteus vulgaris. Biometals. 2021;34(1):175–196. 10.1007/s10534-020-00271-z 33244683

[34] Sana, SS, Kumbhakar, DV., Pasha, A, Pawar, et al. Crotalaria verrucosa leaf extract mediated synthesis of zinc oxide nanoparticles: Assessment of antimicrobial and anticancer activity. Molecules. 2020;25(21):4896. 10.3390/molecules25214896 33113894 PMC7660202

[35] Dey, S, Mohanty, DL, Divya, N, et al. A critical review on zinc oxide nanoparticles: Synthesis, properties and biomedical applications. Intelligent Pharmacy. 2024;3(1):53–70. 10.1016/j.ipha.2024.08.004

[36] Amer, D. A. A., El-Lessy, F. M., Barakat, A. M, et al. Therapeutic Effect of Allium sativum (Garlic) Extract Using Nanotechnology on Murine Chronic Toxoplasmosis. Acta Parasitologica. 2025;70(6):223. 10.1007/s11686-025-01142-8 41238957 PMC12618351

[37] Al-Saadi HK, Awad HA, Saltan ZS, Hasoon BA, Abdulwahab AI, Al-azawi KF, Al-Rubaii BAL (2023) Antioxidant and antibacterial activities of Allium sativum ethanol extract and silver nanoparticles. Trop J Nat Prod Res 7(6): 3105–3110 10.26538/tjnpr/v7i6.5

[38] Bharkavi, SI, Mohideen, K, Sivapathasundharam, B, Murali, P, Femi, AD, Rajendran, R. Antibacterial assessment of Allium sativum against oral pathogens–an in vitro study. Annals of African Medicine. 2025: 10–4103. DOI: 10.4103/aam.aam_549_25 41288553

[39] Bharkavi, S, Mohideen, K, Sivapathasundharam, B, Murali, P., Femi, A, Rajendran, R. Antibacterial Assessment of Allium sativum against Oral Pathogens–An in vitro Study. Annals of African Medicine. 2025: 10–4103. 10.4103/aam.aam_549_25 41288553

[40] Esmaeili, N, Abedian Kenari, A, Rombenso, AN. Effects of fish meal replacement with meat and bone meal using garlic (Allium sativum) powder on growth, feeding, digestive enzymes and apparent digestibility of nutrients and fatty acids in juvenile rainbow trout (Oncorhynchus mykiss). Aquaculture Nutrition. 2017;23(6):1225–1234. 10.1111/anu.12491

